# Encoding of locomotion kinematics in the mouse cerebellum

**DOI:** 10.1371/journal.pone.0203900

**Published:** 2018-09-13

**Authors:** Tomaso Muzzu, Susanna Mitolo, Giuseppe P. Gava, Simon R. Schultz

**Affiliations:** Centre for Neurotechnology and Department of Bioengineering, Imperial College London, London, United Kingdom; Tokyo Medical and Dental University, JAPAN

## Abstract

The cerebellum is involved in coordinating motor behaviour, but how the cerebellar network regulates locomotion is still not well understood. We characterised the activity of putative cerebellar Purkinje cells, Golgi cells and mossy fibres in awake mice engaged in an active locomotion task, using high-density silicon electrode arrays. Analysis of the activity of over 300 neurons in response to locomotion revealed that the majority of cells (53%) were significantly modulated by phase of the stepping cycle. However, in contrast to studies involving passive locomotion on a treadmill, we found that a high proportion of cells (45%) were tuned to the speed of locomotion, and 19% were tuned to yaw movements. The activity of neurons in the cerebellar vermis provided more information about future speed of locomotion than about past or present speed, suggesting a motor, rather than purely sensory, role. We were able to accurately decode the speed of locomotion with a simple linear algorithm, with only a relatively small number of well-chosen cells needed, irrespective of cell class. Our observations suggest that behavioural state modulates cerebellar sensorimotor integration, and advocate a role for the cerebellar vermis in control of high-level locomotor kinematic parameters such as speed and yaw.

## Introduction

An animal’s survival relies heavily upon its ability to locomote through space. The ethological importance of locomotion is reflected by the large proportion of the central nervous system involved in locomotor activity. One such area is the cerebellum, whose function was long ago established through clinical and lesion studies to be essential for learning and controlling movements [1–3]. Being located, in circuit terms, between higher cortical centres and the periphery, the cerebellum acts as a strategic relay point for sensorimotor integration.

Electrophysiological studies combined with the analysis of simple behaviour provided direct evidence for the role of the cerebellum in locomotor control and learning. The spinocerebellum, the central part of the cerebellum, receives projections from the spinal cerebellar tract neurons which convey peripheral sensory signals and information from the spinal pattern generator [4, 5]. In particular, the medial zone of the spinocerebellum, the vermis, has been identified as the area involved in controlling posture, tone, flexion and extension of limbs [6].

The spinocerebellar tracts, which are part of the locomotion circuitry [7], have been found to be largely preserved across a wide range of animal species, including mice [8–10]. The mouse is a model organism of particular interest due to its suitability for the use of transgenic technology to dissect out the contributions of individual circuit elements. In recent years, the application of transgenic techniques to mouse experiments provided new insights into the neural circuits involved in locomotion [11–13], and the role of the cerebellum in motor and cognitive functions [14–17][18–22].

Observation of neural activity in the cerebellum has revealed that many cerebellar neuron types carry step-related information. Purkinje cells are essential for interlimb coordination, adaptation to external perturbation, and they tend to fire rhythmically with the stepping cycle [18, 23–26]. Although Purkinje cells in the cat were not observed to have substantial modulation of firing rate by the speed of walking on a treadmill [27], it was recently observed that the firing rate of Purkinje cells, averaged within single steps, can be modulated both negatively and positively with locomotion speed in freely running rats [28]. Golgi cells were also shown to discharge rhythmically during locomotion, however they were not modulated by the speed of locomotion [29]. Granule cells and molecular layer interneurons of mice on a a spherical treadmill increased their firing rate during locomotion relative to stationary periods [30, 31], leaving open the question as to whether and how cerebellar neurons are tuned to locomotion speed.

To address these questions, we recorded from populations of neurons in lobules IV-V and VIa of the cerebellar vermis of mice navigating in a virtual reality (VR) environment. We characterised neurons whose activity is specifically modulated by locomotion speed and yaw rotation. We found that their firing rate correlates better with future than with past values of locomotion speed. The combined activity of these neurons linearly decodes locomotor speed with an accuracy proportional to the population size, irrespective of the cell type suggesting that cerebellar activity is modulated by high-level locomotion parameters, and sensorimotor information penetrates all computational stages in the cerebellum.

## Materials and methods

### Virtual reality system

**Fig 1:**
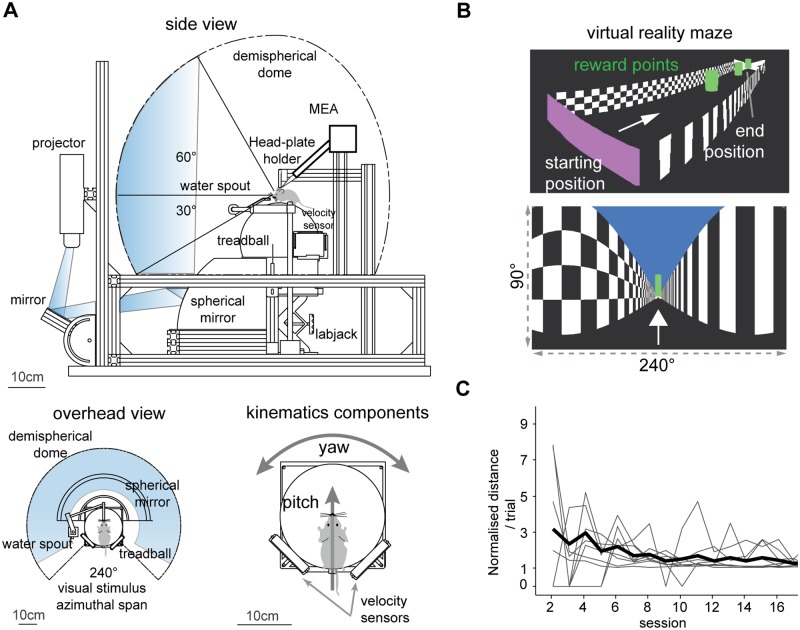
Virtual reality apparatus. (A) Schematic view of the virtual reality system with approximate image path of the visual stimulus from the projector to the demi-spherical screen. Bottom left. Overhead view of the coverage of the mouse visual field. Bottom right. Mouse on the spherical treadmill, or treadball. The signals from the velocity sensors are integrated to determine translation (pitch) and heading (yaw) movements of the mouse in the virtual environment. Mouse drawings not to scale. (B) Virtual reality environment. Top, perspective view of the virtual corridor. Bottom, subject perspective of the VR maze with horizontal and vertical span of the virtual camera. (C) Mean normalised distance ran per trial in first 16 days. Gray lines are subjects, thick black line is average for all trained mice (n = 10).

Experiments were performed in a custom made virtual reality system for mice similar to those used in previous studies [32, 33]. Mice ran on a polystyrene sphere of 20 cm in diameter free floating on a 3D-printed concave inset. The motion of the sphere was read by two USB laser mice (Razer Imperator, Razer Inc, USA) positioned ninety degrees apart on the equator of the sphere. The signals carrying the instantaneous velocities of the sphere were polled at 200Hz by the host computer (Windows 7 OS, Microsoft Corporation, USA) via Labview (National Instruments Corporation, USA). These were then integrated to update the position in the virtual environment. The virtual reality environment was rendered with the openGL API implemented in the C++ language and interfaced with Labview control software via Microsoft dynamic-link libraries (DLL). This was projected via a digital projector (PJD6553w ViewSonic Corporation, USA) onto a demispherical screen around the mouse (Talbot design Ltd, UK) with a refresh rate of 120Hz (Fig 1A). The VR apparatus comprised an automated reward system for water delivery and air puff stimulation controlled via a data acquisition card (NI BNC-2090A and NI PCIe-6321, National Instruments Corporation). Water rewards were delivered to the mouth of the mouse via a 3D-printed water spout connected to a peristaltic pump (Model 80204-0.5, Campden Instruments). Whenever the mouse hit lateral walls of the VR track, low pressure air jets were puffed to its trunk from two lateral copper tubes upon opening of two normally-closed solenoid valves (model PU220AR-01, Shako Co. Ltd) connected to an air pressure regulator. Some experiments were run in the dark by disabling the projection of the visual stimulus and only recording mouse movements on the sphere.

### Surgical procedures

All experiments were performed in accordance with the Animals (Scientific Procedures) Act 1986 (United Kingdom) and Home Office (United Kingdom) approved project and personal licenses. The experiments were approved by the Imperial College Animal Welfare Ethical Review Board under Project License 70/7355. The mice (n = 14, age 16-24 weeks) were housed in the animal facility of Imperial College London under a 12-hour light/dark cycle. All electrophysiological recordings were carried out during the dark phase of the cycle.

Mice were anaesthetised in an induction chamber with 5% isoflurane, and then it was kept at 1.5-2% throughout the surgery. A local injection of 0.5% lidocaine and 0.05% carprofen (Rimadyl, 1 mg/ml) was administered subcutaneously before starting the actual procedure. The fur was trimmed from the mouse head before making a sagittal incision up to the neck. The excess skin was removed and, then, the neck musculature was gently pushed caudally to reveal the interparietal and occipital bone. After that, the skull was cleaned with hydrogen peroxide and then polished from the connective tissue. To create a better grip for the attachment of the head metal plate, the skull bone surface was superficially scrapped with a surgical scalpel. The metal plates were attached to the mouse over the bregma in order to keep the occipital zone clear. For every mouse, the head plate had to be bent and curved to adapted it the skull shape and maximise contact area. It was then attached to the mouse head with a topical skin adhesive (Histoacryl, B.Braun Medical Ltd).

After the head metal plate had been attached, a surgical screw, serving as ground connector (M1 x 2 slot cheese machine screw, Precison Technology Supplies), was fitted into a small craniotomy on one of the parietal bones of the cranium. A mini socket (ED90265-ND, ED8250-ND, Digi-Keys electronics, USA) was attached to the screw head for connecting with the ground wires of the electrophysiology recording system. After that, a plastic well of diameter 3-4 mm was positioned in the central part of the occipital bone on the edge with the parietal bones, aligned with the sagittal suture. This was kept in place with a drop of Hystoacril. Next, all the exposed tissue, together with the base of the head metal plate, was covered with dental cement (Kemdent, Associated Dental Products Ltd), leaving only the head of the ground screw and the well above the vermis clear. Here, a craniotomy and durotomy were made through the occipital bone, over lobule VIa, extending ±0.5mm either side of the midline. This last step was carried out during the first surgery for naïve mice. Otherwise, the craniotomy and durotomy were performed in a second surgery following behavioural training. This was performed at least 24 hours after the water restriction was interrupted to prevent any surgical complications. Briefly, initial procedures of the second surgery were the same as the first one. Once the animal was anesthetised, the coatings of nail varnish were removed with acetone solution, and then the Kwik-Cast and the agarose were gently removed. The part of the occipital bone visible inside the plastic well was cleaned and moisturised before the craniotomy drilling. The dura was also removed only from the area exposed. The craniotomy was covered with phosphate-buffered saline (PBS) and 1.2% agarose. The area above the craniotomy was covered with Kwik-Cast sealant (WPI Ltd, UK) and nail varnish. Animals were allowed to recover at least for 24 hours before the electrophysiology recording (for naïve mice, n = 4) or water restriction and consequent behavioural training began (n = 10).

### Behavioural training

Following a recovery period of at least 24 hours from the surgery, mice were head-fixed on the spherical treadmill for up to 10 minutes for two consecutive days to habituate to the system. Water restriction began on day 2 post-surgery. From day 3, the virtual reality projection was switched on. This is the first session of behavioural training. To motivate participation in the task, a water reward was given through a lick port as the mouse walked underneath cylinders suspended over the corridor (Fig 1B). The water reward was given only at the end of each trial, once the mouse reached the following two criteria: 1) when the mouse was observed to intentionally stop only to lick its water reward and 2) when the mouse could reach the end of the virtual corridor without running more than twice the length of the corridor throughout the trials. Corridor length was progressively increased up to 500 cm depending on the mouse performance and motivation (Fig 1C). Two lateral air-puffers were used to prevent the mouse from hitting the virtual walls of the corridor. They pointed to the rear part of the trunk of the mouse on either side.

One to two hours after the end of a training session we gave the mice water *ad libitum* for at least 30 minutes. Mouse weight was monitored daily as to guarantee that the each animal did not lose more than 20% of its pre-training body weight.

Mice were trained every day for at least 2 weeks and until they were capable of running 20 consecutive trials in under 30 minutes from the start of the session for two consecutive days.

### Electrophysiological recordings

After a 24-hours recovery from the second surgery for trained mice (or from the first surgery for naïve mice), the mice were head-fixed on the spherical treadmill, the craniotomy was exposed after gently removing all layers of nail varnish, Kwik-Cast and agarose. The electrode was inserted at a 45 degree angle along the coronal plane and allowed to stabilize in the cerebellum for approximately 10 minutes once good spike signals were detected. To record activity from lobules IV-V and VIa of the cerebellum, we used 4-shank, 32-channel multielectrode array probes (MEAs) (Neuronexus Technologies, USA, probe model Buzsaki32). Behaviour and electrophysiological activity were then recorded in parallel while the mice navigated in the virtual reality environment.

To maximise mechanical and electrical stability during the electrophysiological recording, the water spout was removed from the mouth of the mouse and airpuffers were diverted from the mouse trunk. We did not observe behavioural changes, as the mice were fully hydrated beforehand, and the airpuff noise was a stimulus strong enough to elicit trajectory corrections. Multiple recordings were acquired from each mouse (39 recordings in total, 310 units; minimum duration 270 seconds, max duration 1833 seconds). The recording sessions lasted up to 50 minutes.

### Data analysis

#### Spike sorting and clustering

Electophysiological data from each shank were processed independently with the *SpikeDetekt* / *KlustaKwik* / *Klustaviewa* software suite [34]. After the spike times were detected and sorted with the automated program, we ‘curated’ the outcome of the spike sorting with the built-in *KlustaViewa* program. At this stage, we selected the units with the highest clustering quality. We kept units that had a central portion of the auto-correlogram completely clean [35]. We merged units that were separated due to shift of the signal in different channels with the help of the clustering features viewing tool. These were also validated against each other by means of the cross-correlograms.

#### Cell classification

The recording electrodes were slowly inserted into the cerebellar cortex until we reached a layer characterised by a “bee-hive” background activity sound on the audio monitor, and the presence of multiple units, distinguishing the granular/Purkinje cell layer from the relatively silent molecular layer [36, 37]. We therefore assume that our recordings are likely to have been made predominantly from the granular and Purkinje cell layers.

**Fig 2:**
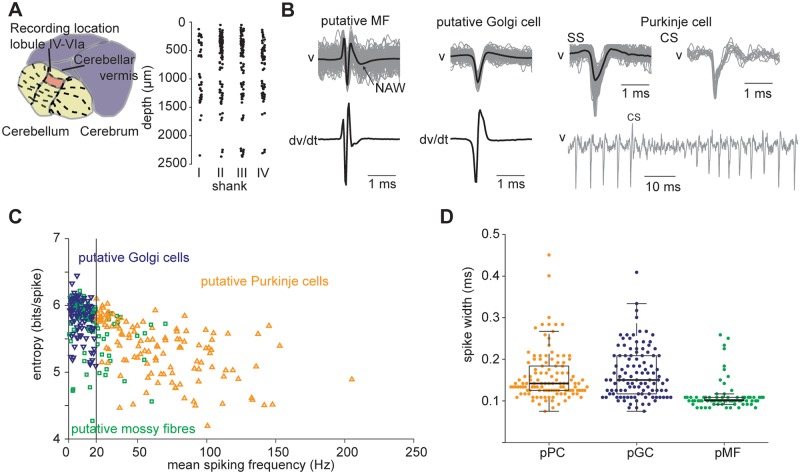
Cell classification of cerebellar units. (A) Left: recording location area. Right: depth of single units (n = 310) from the cerebellar surface grouped by shank (I-IV) for all mice. Depth measurements are based on the position of the channel in which the greatest spike amplitude of the signal is recorded. (B) Examples of action potential waveforms for a putative mossy fibre, Golgi cell and Purkinje cell. Gray traces are single waveforms (100 randomly chosen spike events). The spike duration was computed by estimating the distance between the peaks of the first derivative of the mean action potential trace—shown for the putative mossy fibre and Golgi cell examples. Complex spike mean waveform is averaged from twelve action potentials. Note the pause in simple spiking following the complex spikes. (C) Entropy and mean spiking frequency computed during stationary periods of all units. Black line shows threshold for putative Purkinje cell/Golgi cell classification. (D) Waveform durations of the three classes of putative Purkinje cells (pPC, n = 122), Golgi cells (pGC, n = 111) and mossy fibres (pMF, n = 77).

First, we classified as putative mossy fibres those units that had a short negative waveform preceded and followed by a positive waveform (three-phasic action potential) with a consecutive negative after-wave (Fig 2B). This characteristic waveform shape has been ascribed to mossy fibre glomeruli [36–39].

For the classification of the other units, we used the classification criteria based on a recently published algorithm: two measures of the irregularity of spontaneous activity, namely the entropy of the logarithmic inter-spike intervals (log-interval-entropy), and the mean spiking frequency [40]. For this purpose, spontaneous activity was defined as activity during stationary periods, i.e. when the speed of the mouse was smaller than 1 cm/s. Units were classified if it was possible to extract at least 60 inter-spike intervals (ISIs) from periods of spontaneous activity lasting at least 2.5 seconds. Periods within 0.5 seconds of movement onsets/offsets were removed from the analysis to avoid the effects of preparatory/stabilising movements which did not produce actual motion of the treadmill. As thresholds for the unit classification we adopted the same values used by Van Dijck et al., 2013: units whose mean firing rate exceeded 20 Hz were identified as putative Purkinje cells (n = 122) whereas units with low spontaneous firing rate (<20 Hz) were identified as Golgi cells (n = 111) if the log-interval-entropy was larger than 5 bits (Fig 2C). We manually inspected the raw traces for the presence of complex spikes (CS) to verify the identity of Purkinje cells. Since we used planar silicon multi-electrode array probes, the characteristic CS after-wave was not always apparent in every cell, however, this enabled us to gain confidence in the performance that the algorithm was correctly classifying those cells (n = 16) in which it was possible to identify the CS after-wave and corresponding simple-spike pause (as shown in Fig 2B).

Unit identity was further assessed by comparing the waveform durations of the different putative cell classes. We used this metric as a means to determine whether a unit could be considered as a regular or slow spiking neuron. These types of spiking profiles have been reported to be consistent with mossy fibre and Golgi cell spiking activity respectively (Van Khan et al., 1993). Therefore, we measured the duration of the main negative waveform as the distance between the two peaks of the first derivative of the mean action potential [41]. This approach is equivalent to measuring the width of the main negative waveform at half maximum. We then compared the durations between the putative classes (Fig 2D), finding a significant difference between the putative Golgi cell and putative mossy fibre waveform duration distributions (Mann-Whitney U test, p = 5*e*^−14^) and between the putative Purkinje cell and putative mossy fibre durations (Mann-Whitney U test, p = 1*e*^−15^).

The automated classification process did not identify any units as granule cells. Indeed, the silicon probes we used may not be capable of stably detecting action potentials from their small somata. It is possible that some units could be unipolar brush cells or Lugaro cells, but we could not classify these with our preparation. In fact, there has been no characterisation of these using extracellular signals in vivo, and these cells are less numerous than Golgi cells.

It is noteworthy that the classification algorithm of Van Dijck et al., 2013 was based on data from the cerebellum of anaesthetised rats or decerebrate mice, whereas our experimental preparation uses awake, head-fixed mice on a sphere floating on a cushion of air. This might explain the similar values of entropy and mean spiking frequency of putative mossy fibres and Purkinje cells that we observed, as well the upward shift in entropy, similar to that observed by Dugue et al., 2017 (Figure 1 supplement 3E) or by Van Dijck et al., 2013 (Figure 5C). For this reason we employed a more complete strategy, i.e. presence of at least a CS, waveform shape and spike width measures, to tentatively assign a cerebellar cell class to our units. As ground truth for cell identity is not available, cell classification necessarily remains putative.

#### Tuning curves

Firing rate was sampled at the same frequency as the speed (5 ms bins) and then smoothed with a 150 ms Gaussian filter. The two quantities were then sorted in ascending order of locomotion speed. For speeds greater than 1 cm/s, we sequentially averaged the firing rate and speed values of 2000 bins to estimate each data point of the tuning curve. The data points for speed = 0 cm/s are formed from all bins taken when the mouse is still. To evaluate the significance of a unit’s firing rate modulation with speed of locomotion, we randomly shifted the spike times one hundred times by at least 20 seconds and up to the duration of the recording minus 20 seconds [42]. For each iteration, firing rate was calculated and a speed tuning curve computed, and its variance was measured. We then compared the variance of the original speed tuning curve with the ones from the shuffled data. If its value was greater than 99% of the shuffled data values, then we considered the unit as significantly sensitive to movement (binary response). We repeated this calculation and applied the same criteria to the tuning curves only including speeds ≥1 cm/s to assess whether the firing rate was significantly modulated by locomotion speed and not only by still-moving transitions [43].

A unit response type was defined according to the curve that best fit the original data points. Because of the different response profiles obtained from the original data, three different curves were fitted (linear, second-degree polynomial and double exponential, *R*^2^ = 0.8±0.01, mean±sem, median = 0.87, n = 310). The inverse of the variance of each data point was used as weight for the fitting to compensate for the different number of data points in each bin at speed = 0 cm/s. The coefficients of the best fit curve were used to determine the response type. In addition, we classified a cell as:

- positively modulated if the maximum firing rate was greater than the firing rate during stationary periods, and this was recorded at a speed greater than 70% of the maximum speed of the mouse;
- negatively modulated if the minimum firing rate was smaller than the firing rate during stationary periods, and it was recorded at a speed greater than 70% of maximum speed of the mouse
- having a preferred speed if the maximum firing was greater than the firing rate during stationary periods and this was recorded at a speed smaller than 70% of the maximum speed of the mouse.

The tuning curves for yaw movement were calculated similarly for clockwise (CW) and counterclockwise (CCW) turning of the sphere. We then fitted three different curves (linear, second-degree polynomial and double exponential), selected the best fitting, and calculated the modulation index for either yaw direction. Modulation indexes were calculated as:

$$\begin{array}{c} {\text{Mod}}_{i}=\frac{yaw_{max}-yaw_{min}}{yaw_{max}+yaw_{min}}. \end{array}$$ (1)

We also calculated the difference in Modulation Index between the CW and CCW direction to assess the asymmetry of tuning curves as: Delta Modulation Index = Modulation Index(CW) − Modulation Index(CCW). Cells with a Delta larger than 0.2 were apparently asymmetric on visual inspection. This value was thus used as the effect magnitude threshold for further analysis of yaw-tuned cells.

#### Step cycle modulation

To look at the modulation with stepping cycle, the pitch velocity signal was high-pass filtered at 3 Hz to remove the effect of changes in locomotor speed. The Hilbert transform was then computed and its phase extracted as a function of time. To ensure that pitch velocity changes were due to stepping, only putative stepping cycles longer than 50 ms and occurring only during moving periods (speed ≥1 cm/s) were considered. Each cycle duration was normalised with respect to time and divided in 36 equal intervals. For each interval, the instantaneous firing rate was computed.

Because of the binning of each cycle, step phase modulation was tested for uniformity with the *χ*^2^ test of uniformity [44]. The mean direction *θ* (in radians) of the firing rate distribution of a cell around the step cycle was computed as:

$$\begin{array}{c} \theta =\text{arctan}(\frac{\sum_{i=1}^{n}\frac{\sin\alpha }{n}}{\sum_{i=1}^{n}\frac{\cos\alpha }{n}}) \end{array}$$ (2)

where the numerator and denominator are the mean rectangular coordinates of the resulting phase angle, *X* and *Y* respectively, *α* is the phase angle of the resultant vector $R=\sqrt{X^{2}+Y^{2}}$ for each cycle, and *n* is the number of cycles or steps.

We also calculated the circular standard deviation *σ*, which is a measure of the spread of the firing rate around the mean phase direction, and indicates where approximately 66% of the data lie [45], as $\sigma =\sqrt[{}]{-2lnR}$. We calculated the Phase Selectivity Index (PSI). PSI is defined equivalently to the orientation selectivity index described by [46],

$$\begin{array}{c} PSI=1-\frac{\sum_{i=1}^{n}R{\left(\theta_{n}\right)}^{2i\theta_{n}}}{\sum_{i=1}^{n}R\left(\theta_{n}\right)}, \end{array}$$ (3)

where *R*(*θ*) is the magnitude of the firing rate for any given angle *θ* = [0°: 10°: 360°], for each stepping cycle.

#### Naïve vs. trained

In order to verify whether the virtual reality affected the responses to movement kinematics, we compared the population of units acquired from naïve (n of units = 57) or trained (n of units = 253) animals. We used the Mann-Whitney Test to compare the modulation index distributions in the two conditions for speed and yaw. We also compared the difference in yaw modulation index and the phase index.

### Mutual information

The Mutual Information between instantaneous firing rate and kinematic time-courses was computed using a continuous estimator based on the Kraskov, Stögbauer, and Grassberger (GSK) technique [47]. We used the Matlab implementation of the GSK algorithm in the JIDT toolkit [48]. Firing rates and kinematics variables were computed as described above, and fed into the GSK algorithm, returning a mutual information value for each unit. For the step cycle calculation, the mutual information was computed between the firing rate and the phase angles of the Hilbert transform of the pitch velocity. Only periods during movement were considered, and mutual information was estimated for each cycle and then averaged. In this case, firing rate was computed every 5 ms and smoothed with a Gaussian filter of standard deviation 20 ms.

### Decoding

For every chosen experiment, the recorded cells’ spike trains were binned at 5 ms and then convolved with a Gaussian function (*σ* = 50 ms, window width of 3*σ*) to obtain a time series of instantaneous firing rates for each cell. The locomotion speed time series was convolved with the same Gaussian function. We considered all locomotion speeds ≤1 cm/s to be stationary; these were set to 0 cm/s. Both the firing rate and the locomotion speed time series were then normalised to obtain values between 0 and 1.

Only recording sessions with at least 8 units with at least one unit per type—i.e. putative Purkinje cells (pPC), Golgi cells (pGC), and mossy fibres (pMF)—(resulting in inclusion of 10 sessions from 4 mice) were considered, in order to investigate the scaling of decoder performance with ensemble size. To decode, we used an optimal linear estimator (OLE) which weighted and linearly summed the instantaneous firing rate of each neuron in its ensemble, then rectified the summed output. We tested the incorporation of an additional offset term prior to rectification, but found that it did not improve performance on our dataset. The decoder was trained on 80% of each locomotion session, and tested on the remaining 20%—allowing five-fold cross validation. The OLE reconstruction is given by

$$\begin{array}{c} \hat{v}={\left[\hat{R}w\right]}^{+} \end{array}$$ (4)

$$\begin{array}{c} v=Rw, \end{array}$$ (5)

with $v={\left[v_{1}\ldots v_{T_{\text{train}}}\right]}^{T}$ being the measured speed time course vector for the *T*_train_ training data bins, and **R** a matrix whose columns are the firing rates for the training data, with the addition of a column of ones for the *y* intercept. Training the decoder by linear least squares regression is equivalent to solving this equation to find the optimal value of the estimator:

$$\begin{array}{c} w={\left(R^{T}R\right)}^{-1}R^{T}v, \end{array}$$ (6)

where **v** is a column vector containing the locomotion speed values for the training data. The estimated speed is half wave rectified to reflect the fact that only positive speed values are possible. We assessed decoding performance by computing the Pearson correlation coefficient between the actual and reconstructed locomotion speed time-courses, for the test data.

## Results

### Cerebellar neurons respond to speed of locomotion

**Fig 3:**
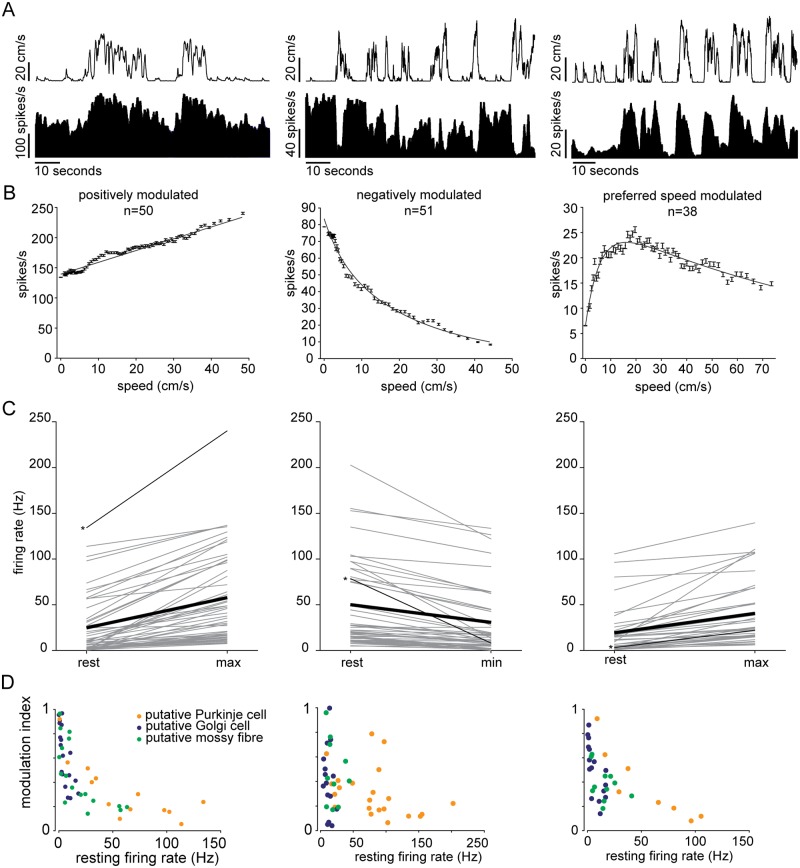
Cerebellar neuronal response to locomotion speed. (A) Speed and instantaneous firing rate of three example units for the three response profiles observed (bins = 0.5 seconds). (B) Speed tuning curves of the examples in A; error bars are standard errors. (C) Mean firing rate during stationary periods and speeds at which maximal firing rate is recorded. Gray lines are single units; thin black lines with asterisks on the left indicate units shown in A and B; thick black lines are average firing rates of all units within each response class. (D) Modulation indexes shown as a function of resting firing rate (i.e. during stationary periods), for each response type.

The activity of many units correlated with the behavioural status of the mouse, i.e. the firing rate changed with speed (Fig 3A). To determine whether neural activity correlated with locomotion speed, we computed the speed tuning curves for the firing rate of each unit (Fig 3B), assessing their significance by shuffling as described in the methods. We also checked whether the changes in firing rate were due only to changes in excitability between stationary and moving periods (i.e. if cells were driven by locomotion, but their activity was not modulated by speed) by repeating the above procedure but only considering speeds >1 cm/s. Of the 310 units recorded, 159 units were found to respond to locomotion state: 20 showed a binary response to stationary-to-locomoting state and the remaining 139 were modulated by speed. For these units, three classes of modulation profile (tuning class) were observed (Fig 3B): units whose firing rate monotonically increased (n = 50) or decreased (n = 51) with speed, and units whose firing rate reached its maximum at a preferred speed that is ≤ 70% the maximal speed achieved by the mouse (n = 38). Similar profile responses were observed in naïve (untrained) mice and no differences were found between the responses of units recorded from these and trained animals. These units were therefore analysed conjointly.

Positively modulated units, on average, had lower resting firing rates compared to those cells whose firing rate decreased with speed (Fig 3C). Firing rate changed from 24.7±4.6 Hz during resting periods to 52.2±7 Hz (mean±s.e.m., n = 50) at maximal locomotion speed. For negatively modulated units, firing rate decreased from 50±7.6 Hz under the resting condition to 30±6.1 Hz (mean±s.e.m., n = 51) during locomotion at maximal speed. Units showing a preferred speed had on average less marked changes going from 19.3±4.2 Hz at rest up to 40±5.7 Hz (mean±s.e.m., n = 38) at the maximum speed observed (Fig 3C).

We examined whether the modulation of single cells differed within and across response tuning classes (Fig 3D). The modulation index was defined as the ratio between the difference and the sum of the maximum and minimum firing rates measured on each tuning curve. Modulation indexes for all response types varied heterogeneously across the whole range. We found that, for all response tuning classes, the modulation of firing rate appeared to be negatively correlated with resting firing rate (Fig 3D).

Units responsive to movement belonged to all putative cerebellar cells classes (Fig 3D) and were observed in all animals, with no discernible dependence on the depth of the recording site. Moreover, units belonging to the same response tuning class were not observed to cluster spatially: only in 8 out of 39 recordings did we find units belonging to the same response class in close proximity (i.e. in the same electrode shank). The response types in the rest of the recordings with closely located multiple units were heterogeneous.

The majority of cerebellar vermis neurons recorded, irrespective of putative cell class, responded to locomotion speed by either increasing or decreasing their firing rate from rest, in some cases responding maximally at a particular preferred speed of locomotion.

### A subset of cerebellar neurons display yaw direction tuned responses

**Fig 4:**
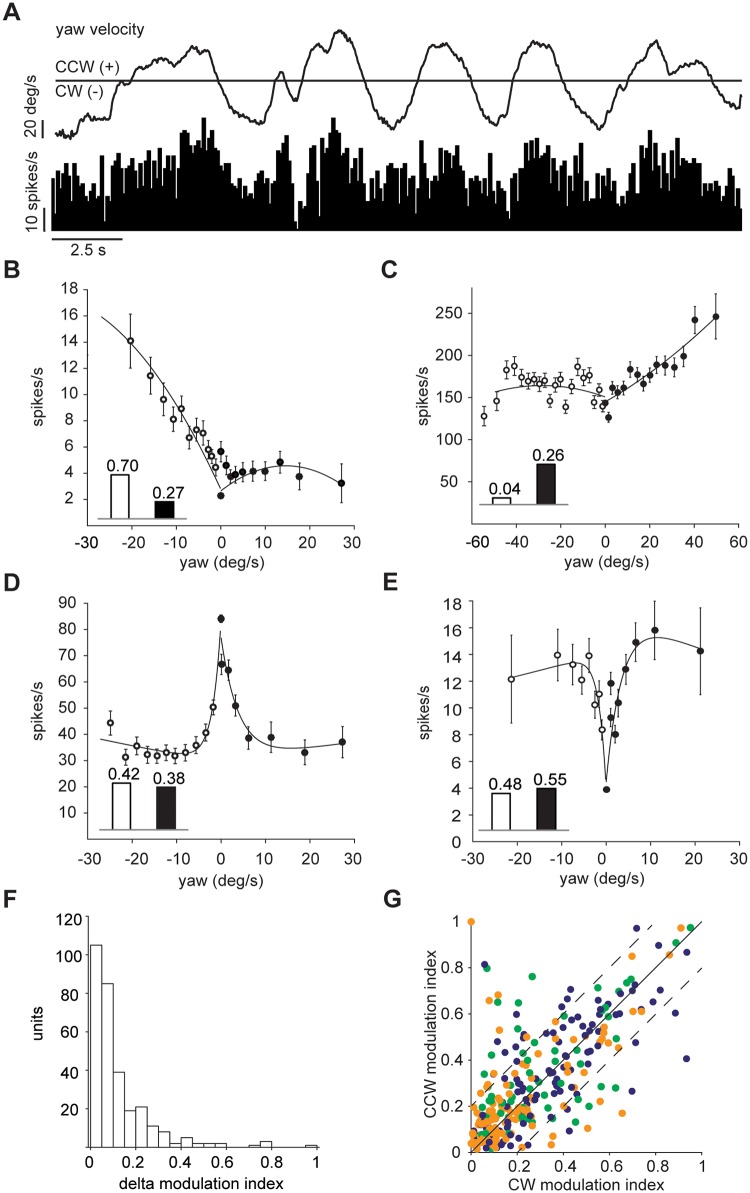
Cerebellar neuronal response to yaw direction. (A) Example unit tuned to CCW yaw direction (positive): top, yaw trace in deg/s; bottom, mean instantaneous firing rate, bin width 100 ms. (B-C) Two example units preferentially responding to yaw-turning in the CW (B) or CCW (C) directions; each data point is formed from at least 900 bins (100ms width); error bars are s.e.m.; inset bars indicate the modulation index values derived from the two curves. (D) Example unit decreasing its activity in both yaw directions. (E) Example unit that increases its activity in either yaw direction. (F) Distribution of the difference in modulation index between the CW and CCW yaw direction. (G) Population plot of modulation index values of each unit for the CW and CCW direction (n = 310). Dashed lines show the threshold for units with difference in modulation index between the CW and CCW yaw direction larger than 0.2. Dot colour indicates cell class as in Fig 2.

While animals ran in the virtual corridor, they corrected their trajectories repeatedly to reach the target location marking the end of each trial. As the animals exerted more strength on either one or the other side of the body when turning the sphere, we examined whether this asymmetric use of limb muscles was reflected in cerebellar neuronal activity. By extracting the yaw movement information from the motion sensors, we examined how the firing rate changed with respect to sphere rotations around the clockwise (CW, negative yaw) and counter-clockwise (CCW, positive yaw) direction (Fig 4A).

To do so, we computed tuning curves for neural activity in response to CW and CCW yaw directions, and calculated the modulation indexes for each cell in the two directions. For a few units, the neural response was clearly one-sided (Fig 4A). This was reflected in their tuning curves, and in the absolute difference between the modulation indexes of the tuning curves computed for the negative (CW) and positive (CCW) yaw directions (delta yaw modulation index—Fig 4B and 4C). Most cells, however, responded equally to either yaw direction, showing a decrease (Fig 4D) or increase (Fig 4E) in firing rate to either CW or CCW yaw movement, in an almost symmetric fashion. This is reflected in the distribution of the delta yaw modulation indexes: 63% (195 out of 310) had a difference in modulation indexes smaller than 0.1, while only 19% (58 out of 310) had greater than 0.2 (Fig 4F and 4G), indicating sensitivity to yaw direction. Many units had similar modulation indexes for both the CW and CCW yaw directions, with no discernible differences between putative cerebellar cell classes (Fig 4G). We also examined whether tuning to locomotion speed influenced the yaw-direction selectivity of the 58 cells that had delta yaw modulation index greater than 0.2. Interestingly, 32 out of the 58 yaw direction sensitive units did not modulate their activity with speed. Indeed, we could find no evident relationship between the speed modulation and delta yaw modulation index.

These results suggest that there are units in the cerebellum that respond selectively to either right or left direction of yaw movement, and that these cells are not necessarily influenced by changes in overall locomotion speed.

### A subset of cerebellar neurons are tuned to phase of stepping cycle

**Fig 5:**
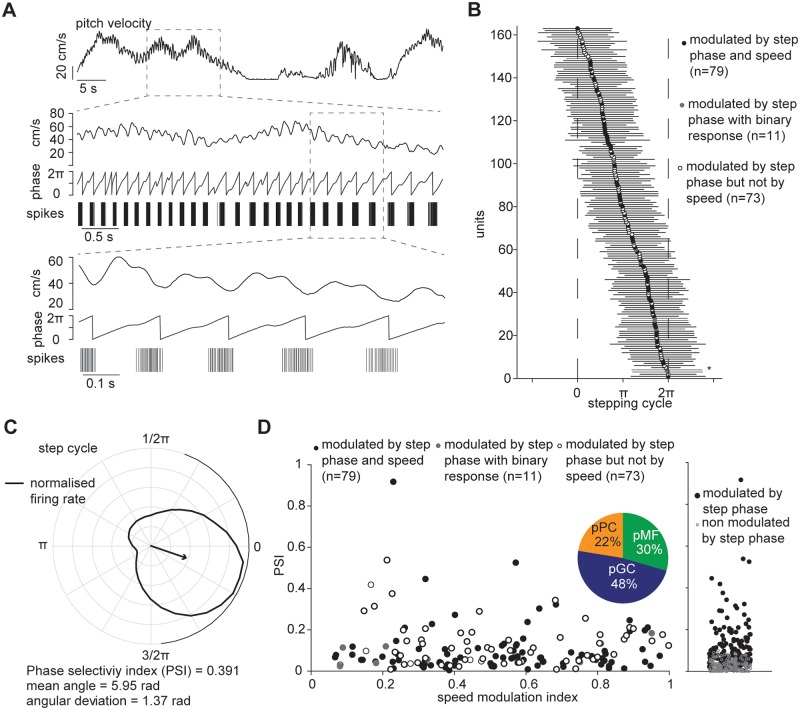
Cerebellar neuronal response to stepping cycle. (A) Top: pitch velocity signal; centre: boxed pitch velocity signal aligned with its Hilbert transformed signal (middle) and spike events of an example unit; bottom: close-up of approximately one second. (B) Preferred phases of units significantly modulated by stepping cycle (n = 163); horizontal bars indicate standard circular deviations. Unit 5, shown in A and C is marked by the asterisk. (C) Polar plot showing the preferred locomotion phase of normalised firing rate for the same unit shown in A; arrow indicates mean angle direction and phase selectivity index, PSI (magnitude of mean response); black solid line around the circle is the circular standard deviation. (D) PSI of units modulated by step phase as a function of speed modulation index. Inset, pie chart of putative cerebellar neurons modulated by stepping phase; pPC: putative Purkinje cells; pCG: putative Golgi cells; pMF: putative mossy fibres. Right, distribution of PSI of these units.

Previous studies that looked at cerebellar activity during locomotion found that Purkinje cells are rhythmically modulated by stepping cycle [24, 25, 27–29, 31]. We reasoned that one way to produce speed tuning would be for units to respond by spiking at a fixed phase per step cycle, thus producing higher firing rates the more step cycles occur per unit of time. We therefore examined whether this was the reason behind the tuning of activity to locomotion speed. Since the pitch velocity components is measured by motion sensors parallel to the vertical axis, with velocity sampled at a high polling rate (f = 200 Hz), it was possible to detect the vertical oscillations caused by the mouse stepping on the sphere (Fig 5A). The high frequency periodicity of the signal was extracted from the original pitch velocity signal by transforming the pitch velocity with the Hilbert operator. On average, there were 712 putative step cycles per recording session (712±119, mean±s.e.m., n = 39), with a mean duration of 0.20s±0.0026 (mean±s.e.m.) and an average stepping frequency of approximately five steps per second. We then measured the correlation between the firing rate (bin width = 5ms, smoothed with a 20-ms Gaussian window) and the phase of the step cycle for each unit (Fig 5B) finding that 163 units out of 310 (53%) showed significant modulation with the stepping cycle (p≤0.01, *χ*^2^ test for uniformity). Of these, only 79 units were also significantly modulated by speed. Mean preferred phases of the modulated units were distributed across the stepping cycle, with approximately two-thirds of the response covering on average 2.2±0.06 (mean±s.e.m, n = 57) radians, as measured by the standard circular deviation [45] (Fig 5B).

To quantify the step phase modulation of the response, we used an approach commonly used in the study of orientation tuning, an analogous problem (where here phase within a step cycle replaces orientation within a circular stimulus space). We calculated the normalised phase orientation vector and computed the orientation selectivity index [46], renamed here the phase selectivity index, PSI. Units significantly modulated by step phase have higher phase selectivity indexes in comparison to non-modulated units (p = 2e^−16^, Mann-Whitney U-test, Fig 5D). This, however, did not correlate with the degree of modulation of the speed tuning curve of the units modulated by stepping phase suggesting that the response to locomotion speed of cerebellar neurons may be driven independently of the rhythmic modulation of the stepping cycle.

### Cerebellar neurons mainly encode motor information

**Fig 6:**
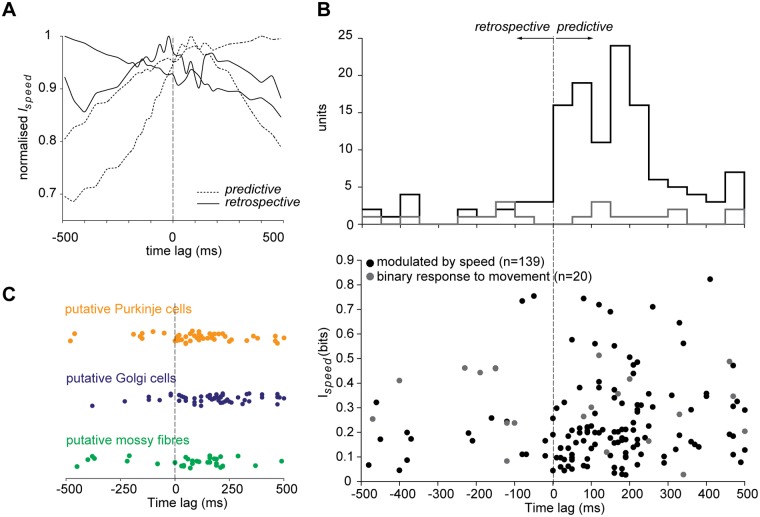
Mutual information between locomotion speed and firing rate. (A) Normalised mutual information for four example units for different time lags; predictive units (maximum mutual information occurring at lag > 0) are indicated by dashed lines, and retrospective units (optimal delay < 0) by solid lines. For each type, two examples are shown: one that either increases or decreases with time and one with a peak either before or after zero lag. (B) Top: Histogram of the distribution of the peaks at which maximal mutual information is found for units responsive to movement and modulated by speed (bin size 50 ms). Bottom: maximal mutual information values of the units responsive to movement and modulated by speed with respect to the time lag. (C) Time lags at which maximal mutual information peaks for the different putative cerebellar cell classes.

In order to understand whether the recorded units encode information about descending motor commands or sensory feedback, we analysed the timing of the information conveyed by neuronal responses about locomotion speed, reasoning that, for motor units, the firing rate should provide predictive information about locomotion speed, whereas for sensory units, the information should be largely retrospective. We computed the mutual information [49] between the firing rate and locomotion speed time courses for each neuron, for a range of imposed time lags. Firing rates were shifted with respect to the speed signal by 10 ms from -500 to +500 ms (Fig 6A). While there were retrospective units (peak mutual information at negative time lag), the majority of speed modulated units showed a peak in the mutual information at a positive time lag of 108.1±17.2 ms (mean±s.e.m., n = 139), suggesting that the neurons recorded, irrespective of cerebellar class, may primarily receive descending motor signals rather than sensory feedback (Fig 6B and 6C).

### Cerebellar units compute multiple kinematic parameters

We have described units tuned for speed of locomotion, yaw (including some tuned for direction of yaw motion), and phase of stepping cycle. It is important to determine whether these constitute separate classes of neurons, or if instead, each of the neurons displays tuning to a lesser or greater extent across each dimension.

**Fig 7:**
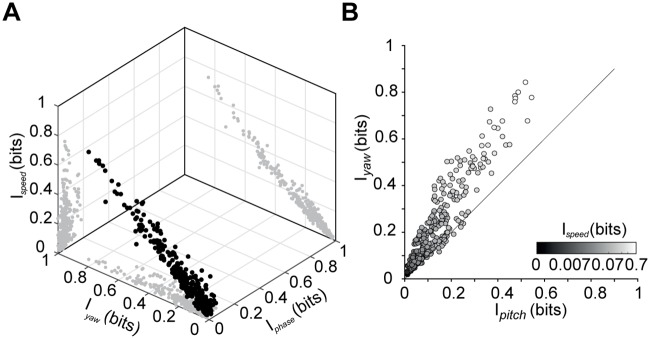
Mutual information between firing rate time series and kinematic parameters. (A) Information about speed, yaw and phase within the step cycle, for each cell (n = 310). Black points plot the three-dimensional joint distribution; gray points show two-dimensional marginals. (B) Mutual information values of the two vectorial components (pitch and yaw) of speed. Grayscale indicates the value of mutual information between firing rate and speed for each cell (n = 310).

We therefore compared the mutual information of all cells recorded about speed, yaw (see Methods), and phase of the stepping cycle. These are depicted in a tri-plot in Fig 7A. It is apparent that speed, yaw and phase selective units do not cluster in this space, but are instead distributed relatively uniformly. As speed and yaw are not independent—indeed, speed is comprised of both a pitch and a yaw component—we broke speed tuning up into these components and assessed them separately. Fig 7B shows that units that conveyed substantial information about pitch also tended to convey substantial information about yaw—and that in fact, more of the speed information arose from yaw than from pitch signals. As bimodal distributions were not found in the information conveyed about speed, pitch, yaw nor step phase, we conclude that the cerebellar units examined were a relatively homogeneous population encoding all of these quantities to a greater or lesser extent, rather than a heterogeneous population comprising clusters of units encoding different kinematic variables.

### Speed of locomotion can be linearly decoded from cerebellar neuronal ensembles

Since the majority of units we found was shown to encode multiple kinematics parameters, we set out to find out whether locomotion speed could be accurately reconstructed by heterogeneous and homogeneous populations of cerebellar neurons.

**Fig 8:**
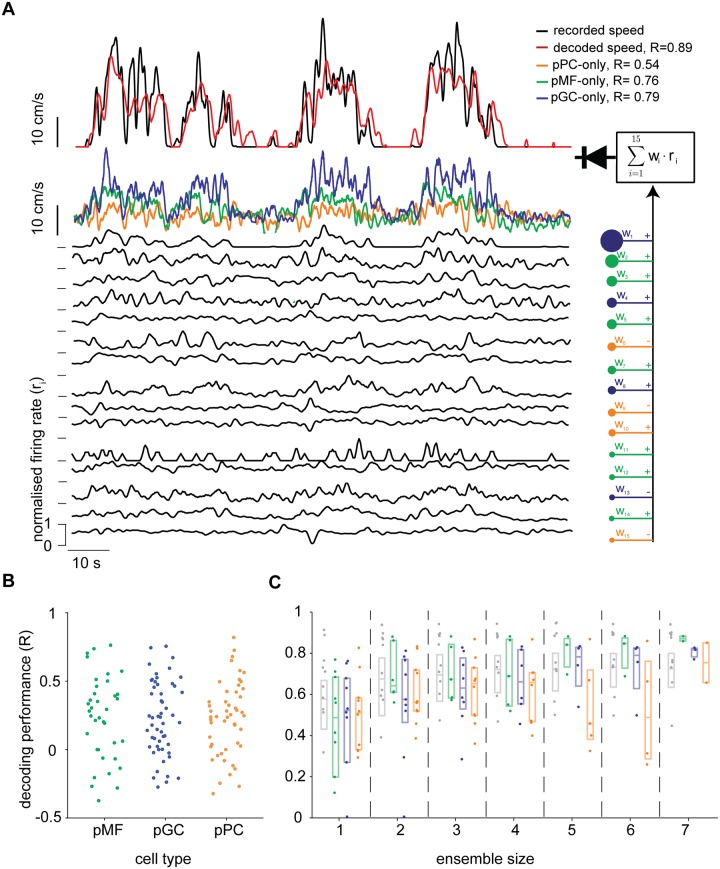
Speed of locomotion can be linearly reconstructed from the activity of a small heterogeneous population of cerebellar neurons. (A) Diagrammatic representation of the optimal linear decoder used to reconstruct locomotion speed, showing one example experiment comprising the three putative cell types (Purkinje cells: pPC; Golgi cells: pGC; mossy figres: pMF). Top: above, time course of original speed signal and decoded trace using the entire population; below, decoded speed traces using only homogeneous ensembles. Bottom: normalised firing rate of all units used for the decoder. Right: firing rates of all units, color-coded according to their putative cellular type, are weighted and linearly summed, then half-wave rectified (diode symbol). (B) Decoding performance for all experiments with at least 8 units recorded, (n = 10). (C) For the same 10 experiments used in panel (B), the scores of the best performing ensembles, selected from random samples (see Results), plotted as a function of the number of units for the selected experiments. For each ensemble size, four different measures are shown: the performance of heterogeneous populations (grey) and of homogeneous ensembles (colour-coded as in panel A).

First, we looked at speed decoding performance of single units to investigate whether there were any differences between putative cerebellar classes and found that there was no apparent difference between these (Fig 8B).

We then investigated how the population size and its composition affected the accuracy of the decoder. To avoid any bias in the choice of units used in the reconstruction, these were selected randomly for any given size of neural population, to then select the best performing ensemble for each population size and type. All possible population permutations were tested for ensembles of 1, 2, *N* − 1 and *N* units (*N* = total number of units recorded in the experiment), and *N*^2^ permutations otherwise. Picking the best performing ensemble, given ensemble size and cellular composition, gives us a meaningful estimate of which cells and combinations the brain could ‘pick’ in a specific decoding situation. We found that a) on average, the decoder performance increases with the ensemble size and b) mossy fibres and Golgi cell ensembles yielded higher decoding accuracy compared to Purkinje cell ones.

These results suggest that most of the cerebellar units, regardless of their type, receive information related to instantaneous locomotion speed but, at population level, the amount of locomotion speed information may depend on their type.

### Response properties do not depend on cortical location

**Fig 9:**
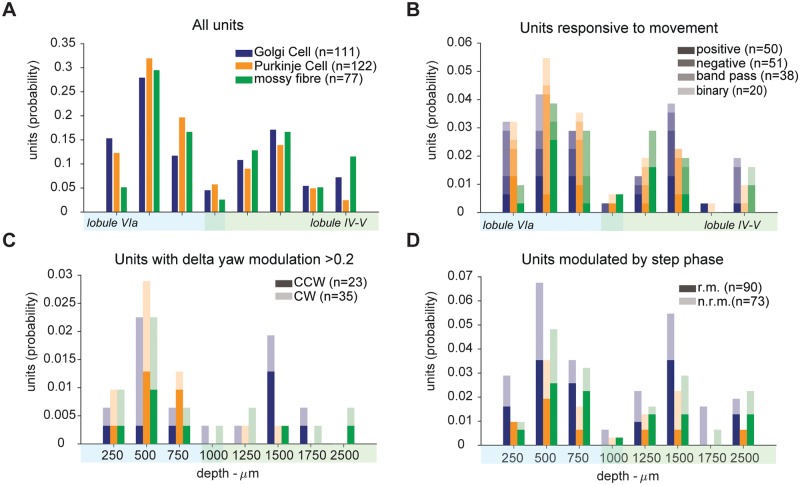
Recording location does not affect response properties. (A) Distribution of recording location for all recorded neurons. (B) Distribution of recording location for neurons responsive to movement. (C) Distribution of recording location for units with a preference for either yaw direction. CCW: counter-clockwise; CW: clockwise. (D) Distribution of recording location for units modulated by step phase responsive to movement (r.m.) and not responsive to movement (n.r.m.). Shaded areas below x axes indicate vermal lobule of recording. Overlap around 1000 *μ*m indicate uncertain lobule identity. Lobular location was deduced based on histology data, see S1 Fig.

We then asked whether the response properties of the neurons we have found depended on their position in the cerebellar cortex. First, we found that cerebellar cells modulated by speed were found in the lobules (IV-V and VIa) from which we made the recordings and they did not show a dependence on the depth of the recording (Fig 9B). Similarly, we found units that showed a preference for either turning (yaw) direction and units modulated by step phase at almost any depth and in both lobules -Fig 9C and 9D). These data indicate that the response properties we characterised do not depend on vermal lobule or recording depth within the anatomical region of observation.

## Discussion

In this study, we used a virtual reality behavioural task, together with multi-unit electrophysiological recording, to investigate the neuronal responses to locomotion. Our recordings from lobules IV-V and VIa of the vermis indicate that most cells in this area are modulated by kinematic parameters of locomotion. We found that while 6% of the cells showed significantly different firing rates during locomotion as opposed to rest, but no significant modulation by speed (similar to the classical results of Armstrong and Edgley 1984, 1988), 45% of neurons were specifically tuned for speed of locomotion. Our data show that such speed modulation is present in cerebellar neurons that are not modulated by step phase. We also found some cells that were tuned to yaw (turning) direction. In general, firing rate of the recorded neurons correlated better with future than with past locomotion speed, suggesting that that these may be driven by an internal signal. At a population level, we found that ensembles of mossy fibres and Golgi cells yielded higher decoding accuracy compared to Purkinje cell ones.

While we have employed and extended recently developed cell classification algorithms [40, 50], our approach is limited by the lack of certainty about cell identity. We were able to putatively identify Purkinje cells, Golgi cells, and mossy fibres based on recording site, waveform characteristics and two irregularity measures of firing rate. To verify the identity of Purkinje cells, we checked for the presence of complex spike (CS) waveforms in the raw traces. Most likely due to the use of silicon probes, the characteristic after-waves of the complex spike was not always visible. For this reason, we excluded complex spike activity from the analysis.

Further improvements to cell classification algorithms will probably require obtaining ground truth validation data through simultaneous MEA and sharp electrode or whole cell patch clamp recording in the awake mouse, with histological validation, a technically challenging task. In our study, however, we did not find the responses to locomotion kinematic parameters to be dependent upon cell class. Indeed, this result is not surprising in view of multiple reports that showed that different cerebellar neurons have very similar responses: Purkinje cells [4, 25, 27, 28], granule cells [31] and Golgi cells [29] have been observed to discharge rhythmically during locomotion suggesting that different cerebellar neurons may encode similar information.

The closed loop VR system for head-fixed mice allowed us to assess the activity of multiple neighbouring neurons of the cerebellum in realistic conditions. Animals can move willingly in a two-dimensional virtual environment with the advantage of controlling for vestibular inputs. At the same time, the use of a (spherical) treadmill makes our results comparable with former studies [27, 29] while keeping technical sophistication to a minimum compared to preparations with chronic implants, with accelerometers for inertial (vestibular) measurements and video recording for speed tracking (e.g. [28]).

Kinematic parameters of arm movements have been found to be related to single neuron activity in the cerebellum [51, 52] and in the motor cortex [53, 54] of non-human primates [36, 55–57]. Sauerbrei et al. (2015) did report speed modulation of some of the cells in their dataset (recorded from freely moving rats), but did not describe speed tuning further, instead focusing on step-phase dependent correlations. Ozden et al., (2012) and Jelitai et al. (2016) reported molecular layer interneuron activity to increase following transition from a resting to a moving (locomotion) state. To the best of our knowledge, the current study is the first description of the tuning of cerebellar neurons to speed of locomotion. One question that may arise is why this was not observed previously, for instance in earlier studies of cats walking on a standard linear treadmill [27]. Firstly, we did not map the cutaneous receptive fields of the cerebellar areas from which we recorded, therefore we did not restrict our attention to cerebellar zones responsible for receiving skin/tactile inputs. Differently to studies focusing on the cerebellar representation of simple motor actions, such as stepping or arm reaching movements, we investigated how a relatively high level variable, locomotion speed, influences the cerebellar neural activity. Secondly, while it is not possible, at this stage, to rule out that inter-species differences account for this, it may be that the passivity of walking on a fixed-speed treadmill or the repetitiveness of reaching movements might not engage the cerebellum sufficiently. Our behavioural paradigm, engaging both motor control and adaptive learning, may have better stimulated or placed more demand upon the cerebellum, resulting in the speed tuning responses observed here. In our study, animals actively locomoted on a spherical treadmill levitating on an air-cushion at speeds of their own choice (starting and stopping as they wished), motivated by an increasing reward rate for more rapid progress down the VR corridor segments, which may engage cerebellar networks to a greater degree. A small amount of speed modulation is apparent on close inspection of Fig 2 of Armstrong and Edgley (1988) for most of the neurons they recorded, suggesting that the situation in the mouse and cat may not be completely dissimilar.

We characterised the response of cells that significantly increased their firing rate with increasing running speed, cells that decreased their firing rate below the spontaneous (rest) level with increasing speed, and cells showing a preferred locomotion speed. The existence of these disparate tuning profiles is puzzling, as these were recorded from all putative cell types and their heterogeneity within small volumes (from channels of a single shank of the recording probe, i.e. ≲200 microns) leave open many questions as to how these responses contribute to sensorimotor integration. A recent study showed that the increase or decrease of activity of Purkinje cells depend on the balance between excitation and inhibition from granule cell inputs and molecular layer interneurons respectively [58]. One possibility is that the cerebellar activity might modulate motor synergies necessary for running at different speeds, such as gait changes (walk, trot, gallop bound [12]) with which distinct networks of subcortical neurons are engaged [59, 60]. Further studies will be required to assess this hypothesis, possibly combining genetic techniques with whole-body video recording during locomotion behaviour [13, 61].

By allowing the spherical treadmill to rotate along any orientation (rather than constraining it to a single axis as with a treadmill), we aimed to create a navigation paradigm congruent with a real-world scenario. Animals had to intentionally engage many muscles relevant for locomotion, and were not constrained to run at fixed speeds. However, the locomotion task should be thought of as a sensorimotor control task, rather than as normal locomotion behaviour [62], because of the artificial nature of the head fixation and of the act of balancing on a frictionless ball, which is itself an acquired skill. Since the animals were head-fixed, we assumed that vestibular inputs were negligible during yaw (turning) movements. The correlation of neuronal activity with the direction of movement may be related to lateralised spinocerebellar inputs or different motor synergies employed to steer clockwise or anti-clockwise. We did not find cells with a preference for a turning direction (i.e. a high delta modulation index) to be highly modulated by speed, suggesting that speed and direction of locomotion information are relayed separately.

According to our information theoretic analysis, the majority of the units provided maximal information about the speed of locomotion a short time in the future (∼100 ms). They can thus be thought of as providing predictive, rather than retrospective, information about locomotion, suggesting that they may be driven by internally generated rather than sensory signals. The cerebellum receives projections from the nuclues cuneiformis [63] that, in turn, receives inputs from the mesencephalic locomotor region (MLR—[64]). Since the MLR is a region of the hind brain that is involved in initiating and modulating locomotion [13, 65, 66], the cerebellum might receive a copy of the motor signals sent to spinal locomotion centres [67]. Indeed, MLR neurons have been observed to show similar speed tuning profiles to those reported here [66]. We believe that this signal could have modulatory effects on sensorimotor processing during locomotion, a common phenomenon observed in other cortical areas [42, 43, 68, 69] as well as across phyla [70, 71]. Cerebellar neurons might change their firing rate to deal with faster motor and sensory processing required during locomotion. We also speculate that the suggested internal origin of this speed signal is in agreement with computational theories, based on forward internal models, according to which the cerebellum is provided with an efference copy to compensate for slow sensory feedback during fast movements [72, 73] and to reduce motor noise during movements [74–76].

All three classes of cerebellar cells were found to be able to decode the animal speed, suggesting that the locomotion speed signal pervades most computational steps in the cerebellum. Indeed, single unity activity could provide an accurate readout of locomotion speeds irrespective of their putative cerebellar cell type. The performance of our decoder increased with population size, as it can be expected, suggesting that incoming motor commands (e.g. signals from MLR or from the central pattern generator) or sensory feedback can be linearly combined to minimise noise of the output signal, at the deep cerebellar nuclei [77–81]. The fact that ensembles of putative Purkinje cells decoded locomotion speed with lower accuracy than mossy fibres’ and Golgi cells’ is not surprising as these represent the first processing stage of incoming signals to the cerebellum, so the locomotion speed signal may be cleaner. Relaying locomotion speed information to multiple cerebellar neurons, across all of its cortical layers, could provide a more accurate estimate of the real speed of the animal and optimise motor control, similarly to what has been described for population decoding of saccades duration in monkeys [82, 83].

Historically, the activity of cerebellar neurons during locomotion has been observed to encode stepping movements whereas here, to the best of our knowledge, we report for the first time speed modulation profiles from neurons in vermal lobules IV-V and VIa. Because of the predictive nature of firing rate with respect to running speed, we argue this signal may carry a motor efference copy coming from higher cortical areas. This interpretation brings two consequences: first, cerebellar computation is constantly modulated by the behavioural state (i.e. running speed) and, second, classic step phase dependency and speed modulation are likely to be driven by two distinct inputs. Here, we show that cerebellar neurons are modulated by yaw (turning) movements, corroborating the hypothesis that the cerebellum controls motor synergies [4]. However, the details of how these information are processed across cerebellar zones and integrated by deep cerebellar nuclei to control motor behaviour remain unclear. The cerebellum is characterised by a peculiar fractured somatotopy with abundant collateral connections [84–90] and a modular divergence-convergence configuration of its inputs and outputs [91, 92] so testing these hypotheses will require use of state-of-the-art transgenic tools for mapping the activation of circuitry during behaviour [93–95].

In addition, since speed modulation of neural activity has been reported in many non-motor cortical areas [42, 43, 68, 96, 97], it will be important to assess whether cerebellar motor estimations/predictions are also relayed to, and exploited by, other nervous centres, to integrate behavioural information relevant for sensory processing and spatial navigation [98]. In future work, we hope that this may be elucidated.

## Supporting information

S1 Fig Estimation of recording location. (A) Left: mouse brain showing insertion mark in the vermal lobule VIa. Right: NeuroNexus ‘Buzsaki32’ probe model. (B) Top: sagittal slice of a mouse brain from Allen Institute database software Brain Explorer 2 with superimposed probe estimated trajectory (red) of the recording probe. Bottom: three coronal slices with superimposed histology slices. Red marks show the probe shanks stained with DiI.
(TIF)
