# Supplementary figures and images for: Encoding of locomotion kinematics in the mouse cerebellum
Source: PLoS One. 2018 Sep 13;13(9):e0203900. doi: 10.1371/journal.pone.0203900 (PMC6136788; doi:10.1371/journal.pone.0203900)

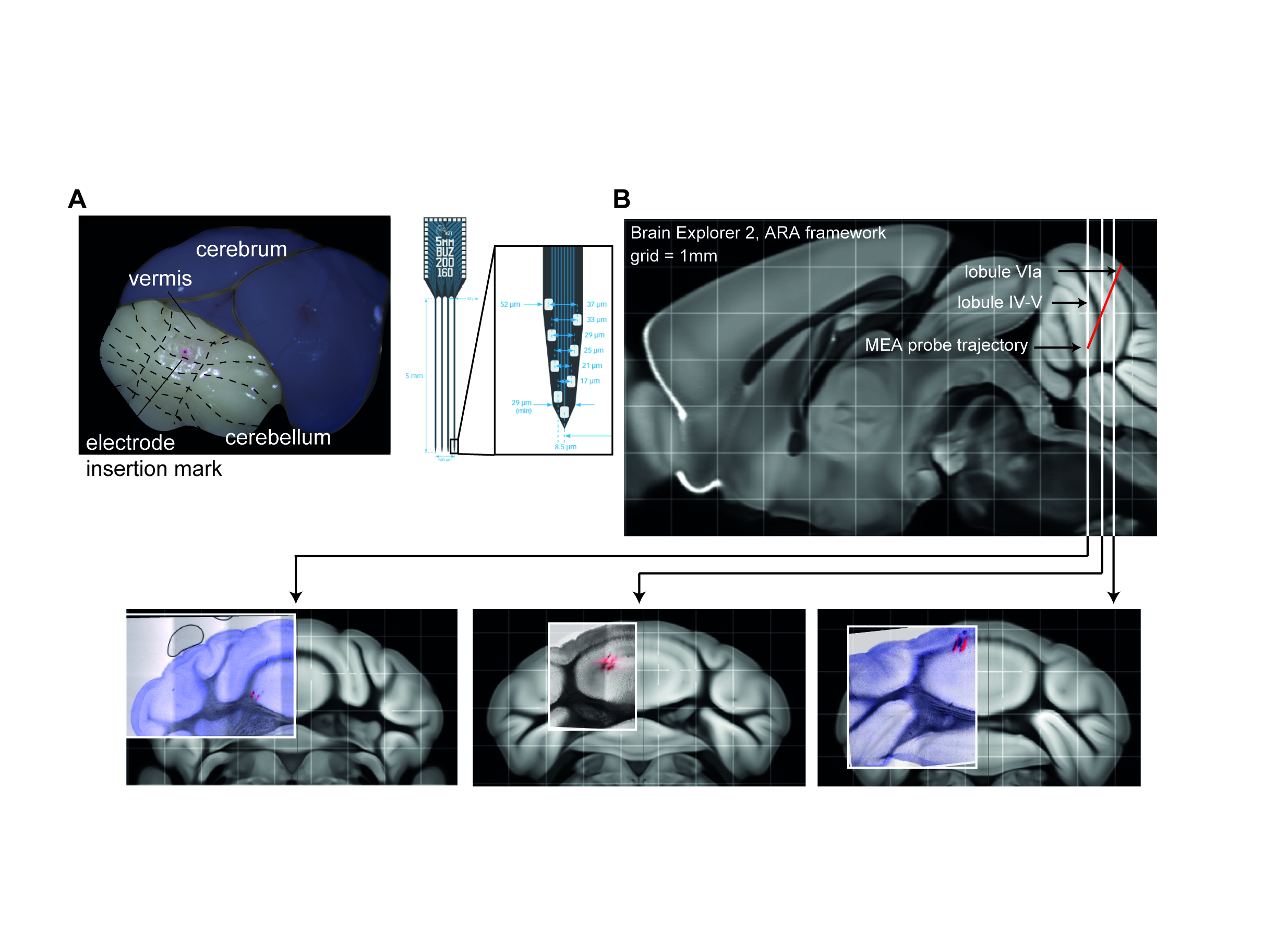

Supplement: S1 Fig — (A) Left: mouse brain showing insertion mark in the vermal lobule VIa. Right: NeuroNexus ‘Buzsaki32’ probe model. (B) Top: sagittal slice of a mouse brain from Allen Institute database software Brain Explorer 2 with superimposed probe estimated trajectory (red) of the recording probe. Bottom: three coronal slices with superimposed histology slices. Red marks show the probe shanks stained with DiI. (TIF) [file pone.0203900.s001.tif]
